# Pancreatic resection for intraductal papillary mucinous neoplasm– a thirteen-year single center experience

**DOI:** 10.1186/s12885-016-2887-8

**Published:** 2016-11-04

**Authors:** Katharina Marsoner, Johannes Haybaeck, Dora Csengeri, James Elvis Waha, Jakob Schagerl, Rainer Langeder, Hans Joerg Mischinger, Peter Kornprat

**Affiliations:** 1 Department of General Surgery, Medical University of Graz, Auenbruggerplatz 29, A-8036 Graz, Austria; 2 Institute of Pathology, Medical University of Graz, Graz, Austria

**Keywords:** Invasive intraductal papillary mucinous neoplasm (IPMN), Non-invasive IPMN, Invasive IPMN, IPMN associated carcinoma, Pancreatic resection, Perioperative outcome, Long-term survival

## Abstract

**Background:**

The purpose of this study is to review our results for pancreatic resection in patients with intraductal papillary mucinous neoplasm (IPMN) with and without associated carcinoma.

**Methods:**

A total of 54 patients undergoing pancreatic resection for IPMN in a single university surgical center (Medical University of Graz) were reviewed retrospectively. Their survival rates were compared to those of patients with pancreatic ductal adenocarcinoma.

**Results:**

Twenty-four patients exhibit non-invasive IPMN and thirty patients invasive IPMN with associated carcinoma. The mean age is 67 (+/-11) years, 43 % female. Surgical strategies include classical or pylorus-preserving Whipple procedure (*n* = 30), distal (*n* = 13) or total pancreatectomy (*n* = 11), and additional portal venous resection in three patients (*n* = 3). Median intensive care stay is three days (range 1 – 87), median in hospital stay is 23 days (range 7 – 87). Thirty-day mortality is 3.7 %. Median follow up is 42 months (range 0 – 127). One-, five- and ten-year overall actuarial survival is 87 %; 84 % and 51 % respectively. Median overall survival is 120 months. Patients with non-invasive IPMN have significantly better survival than patients with invasive IPMN and IPMN-associated carcinoma (*p* < 0.008). In the subgroup of invasive IPMN with associated carcinoma, a positive nodal state, perineural invasion as well as lymphovascular infiltration are associated with poor outcome (*p* < 0.0001; <0.0001 and =0.001, respectively). Elevated CA 19-9(>37 U/l) as well as elevated lipase (>60 U/l) serum levels are associated with unfavorable outcome (*p* = 0.009 and 0.018; respectively). Patients operated for pancreatic ductal adenocarcinoma show significantly shorter long-term survival than patients with IPMN associated carcinoma (*p* = 0.001).

**Conclusions:**

Long-term outcome after pancreatic resection for non-invasive IPMN is excellent. Outcome after resection for invasive IPMN with invasive carcinoma is significantly better than for pancreatic ductal adenocarcinoma. In low- and intermediate risk IPMN with no clear indication for immediate surgical resection, a watchful waiting strategy should be evaluated carefully against surgical treatment individually for each patient.

## Background

The intraductal papillary mucinous neoplasm (IPMN) is the most frequent cystic lesion of the pancreas, originating from the epithelial cells of the pancreatic duct or its branches. IPMN produce mucin and leads to typical dilatation of pancreatic ducts [1]. In 1996, the entity of IPMN was included in the World Health Organization (WHO) classification of pancreatic neoplasms [2]. The histopathological diagnosis of IPMN requires the presence of neoplastic epithelium with intraductal proliferation of columnar mucinous cells, generally with papillary architecture [1, 3]. IPMNs as well as other cystic pancreatic tumors are increasingly diagnosed, mostly due to the greater diagnostic accuracy of radiologic imaging modalities such as multi-detector row computed tomography (MDCT) and magnetic resonance imaging (MRI) and better awareness on the part of pathologists of this entity. Frequently, a cystic pancreatic lesion is diagnosed incidentally in asymptomatic patients undergoing abdominal diagnostics for other potential pathologies [4].

IPMNs are classified according to their radiologic and macroscopic morphologic features into a main duct (MD-IPMN; from 16 – 36 %), a branch duct (BD-IPMN; from 40 – 65 %) and a mixed type (15 – 23 %). The rate of invasive IPMN is significantly higher in main and mixed type lesion than in branch duct IPMN [5, 6]. The malignant potential of IPMN is based on an adenoma-carcinoma sequence [6, 7], which is not the case for ductal adenocarcinoma of the pancreas, where the sequence from low grade to high grade pancreatic intraepithelial neoplasia (PanIN) and further to ductal adenocarcinoma is well established [8]. IPMNs are further classified according to the degree of dysplasia as low, intermediate and high grade dysplasia as well as IPMN with associated carcinoma, previously described as adenoma, borderline and carcinoma in situ and invasive carcinoma lesions [9, 10]. Histopathological and immune-histochemical analysis differentiates between four subtypes: the intestinal, the gastric, the oncocytic and the pancreato-biliary type [11–14]. An IPMN can occur with associated adenocarcinoma as well as concomitant adenocarcinoma, the latter with lower long-term survival [15, 16].

The localization of an IPMN can be uni- or multifocal, and determines the type of surgical resection [2, 6, 7].

The treatment modalities of IPMN were described in the Fukuoka guidelines: patients with main or mixed duct IPMN should be always scheduled for surgical resection. Branch duct lesions with „worrisome features“(cystic size > 30 mm, thickened cystic walls, non-enhanced mural lesions, dilatation of the pancreatic duct of 5 – 9 mm, lymphadenopathy, distal pancreatic atrophy, caliber alterations of the pancreatic duct) or “high-risk stigmata” (duct dilatation > 5 mm, solid enhancing components with biliary obstruction) should be considered for surgical resection. Clinically symptomatic lesions always require surgical intervention [7].

This study reports our experience with pancreatic resection for IPMN in a total of 54 patients over a 13-year period.

## Methods

### Patients and methods

Fifty-four consecutive patients (43 % female; mean age 67 +/- 11 years) who in a 13-year period underwent pancreatic resection for IPMN with or without associated carcinoma at our institution were reviewed retrospectively. Patients with infiltration of portal venous branches requiring vascular resection and subsequent reconstruction were included. Survival rates were compared to a total of 221 patients operated for pancreatic ductal adenocarcinoma during the same period at our institution. The institutional review board approved the study and waived the need for patient consent according to the Helsinki and its own criteria [EK 25 -404 - ex 12/12].

### Preoperative diagnostic algorithm

All patients underwent a detailed clinical examination, blood testing including functional liver parameters as well as tumor markers carcino-embryonic antigen (CEA) and carbohydrate antigen (CA) 19-9. Preoperative radiologic imaging included abdominal ultrasound, MDCT with pancreas protocol and/or MRI with cholangio-pancreaticography (MRCP) and/or endoscopic retrograde cholangiopancreatography (ERCP). Computed-tomography (CT) guided biopsy was performed for further investigation of unclear cystic lesions of the pancreas; it should be noted that during the study period, the newer methods of endoscopic ultrasound (EUS) and fine needle aspiration (FNA) saw increasing clinical application. Positron emission tomography (PET) scan was performed for oncological staging.

### Surgical approach

The type of resection was based on tumor localization: patients presenting with lesions of the pancreatic head or processus uncinatus received standard or pylorus preserving pancreatico-duodenectomy; those with lesions in the pancreatic tail and/or body received distal pancreatectomy with splenectomy in patients with invasive lesions, and spleen-preserving surgery when the intraoperative rapid frozen section showed no invasive component. Total pancreatectomy was conducted in patients with diffuse distribution of IPMN and/or large tumor size involving the pancreatic head and body. The bilio-digestive anastomoses were connected with 5-0 or 6-0 double layer single sutures. Internal drains placed routinely to protect the pancreatico-jejunostomy and the hepatico-jejunostomy, which were both performed as end-to-side anastomosis. The standard protocol called for intraoperative rapid frozen section diagnosis and depending on that histopathologic diagnosis, resection was extended until negative margins could be obtained when there was high-grade dysplasia, invasive IPMN and/or high-grade PANin. If this was not possible, the surgical strategy was changed to total pancreatectomy.

### Follow-up protocol

Postoperative complications were classified according to the system established by Clavien and Dindo [17]. All patients underwent clinical, laboratory and radiological follow-up three, six and twelve months postoperatively, and every six months thereafter. Follow-up data were obtained from the patient’s records, the hospital database and the national cancer registry [Austrian National Cancer Registry; [18]].

### Statistical analysis

Data were acquired prospectively and saved an institutional pancreatic database including baseline data, pre-, intra- and postoperative parameters, results of histopathological and immune-histochemical assays and follow-up details.

Data were collected retrospectively in an Excel database (Microsoft Inc., Redmond, USA) All statistical analyses were performed with SPSS 22.0 for Windows (IBM Inc., Somers, USA). If not otherwise indicated, continuous variables were reported as mean and standard deviation; categorical data were reported as count and percentages. Categorical variables were compared with Fisher’s exact or the Chi-square test, as appropriate; for numeric variables, we used the Wilcoxon test. A *p* < 0.05 was considered statistically significant. Overall survival was calculated according the method of Kaplan and Meier. Differences between subgroups were compared with the log-rank test.

## Results

### Preoperative results

Mean age is 67 ± 11 (range 29 – 84) years, 43 % female, mean body mass index was 25 ± 4. At initial presentation, seventy-eight percent of patients are symptomatic; only 22 % are asymptomatic and diagnosed incidentally with cystic pancreatic mass after undergoing abdominal imaging for other reasons. Seven percent of patients have jaundice with a serum bilirubin value greater than 3 mg/dl. The distribution of baseline and preoperative patient’s characteristics in both non-invasive and invasive subgroups is presented in Table 1. Initial diagnostic imaging always includes abdominal sonography; further, there is MDCT with pancreas protocol in 91 % and abdominal MRI in 69 %. For detailed evaluation of the pancreatic duct system, we perform an ERCP in 39 % and MRCP in 35 %. A malignant lesion is suspected in 20 % and staging diagnostics are completed with a PET scan; CT-guided biopsy is performed in 19 %. In preoperative radiological diagnostic imaging, a cystic diameter > 3 cm is present in 38 % of patients with non-invasive and 47 % of patients with invasive IPMN (*p* = 0.50). Preoperative liver function parameters are displayed in detail in Tables 2 and 3.

**Table 1:** Demographic and preoperative patient data

Factor	Overall (*n* = 54)	Non-invasive IPMN^a^ (*n* = 24)	Invasive IPMN^a^ (*n* = 30)	Two-sided *p*-value
Age (years)	67 ± 11	66 ± 12	67 ± 11	0.70
Age > 70 years	23 (43 %)	9 (38 %)	14 (47 %)	0.50
Female gender	31 (57 %)	14 (58 %)	17 (57 %)	0.90
Body mass index	25 ± 4	26 ± 4	25 ± 4	0.51
*American Society of Anesthesiologists (ASA) classification*				
ASA I	3 (6 %)	3 (13 %)	0	0.06
ASA II	19 (35 %)	8 (33 %)	11 (37 %)	0.86
ASA III	25 (46 %)	11 (46 %)	14 (47 %)	1.00
ASA IV	7 (13 %)	2 (8 %)	5 (17 %)	0.42
*Chronic health factors*				
Smoking	18 (33 %)	8 (33 %)	10 (33 %)	1.00
Alcoholism	7 (13 %)	2 (8 %)	5 (17 %)	0.37
*Preoperative symptoms*				
Abdominal pain	36 (67 %)	15 (63 %)	21 (79 %)	0.57
Nausea	7 (13 %)	1 (4 %)	6 (20 %)	0.09
Diarrhea	3 (6 %)	1 (4 %)	2 (7 %)	0.69
Weight loss	15 (28 %)	5 (21 %)	10 (33 %)	0.32
Diabetes	27 (50 %)	7 (29 %)	20 (77 %)	0.006
Jaundice	4 (7 %)	1 (4 %)	3 (10 %)	0.42
*Comorbidities*				
Arterial hypertension	31 (57 %)	14 (58 %)	15 (50 %)	0.91
Coronary artery disease	8 (15 %)	4 (17 %)	4 (13 %)	0.73
Chronic obstructive pulmonary disease	4 (7 %)	2 (8 %)	2 (7 %)	0.82
Gastroesophageal reflux disease	9 (17 %)	2 (8 %)	7 (23 %)	0.14
Gastritis	2 (4 %)	1 (4 %)	1 (3 %)	0.87
Hiatus hernia	6 (11 %)	2 (8 %)	4 (13 %)	0.56
Extra-pancreatic malignancy (current/anamnestic)	14 (26 %)	6 (25 %)	8 (27 %)	0.89

^a^ *IPMN* intraductal papillary mucinous neoplasm

**Table 2:** Preoperative laboratory tests

Laboratory parameter	Overall cohort (*n* = 54) mean ± SD^a^	Non-invasive IPMN^b^ mean ± SD^a^	Invasive IPMN^b^ mean ± SD^a^	Two-sided *p*-value
Alanin-Aminotransferase (ALT) (Units/liter (U/l))	45.0 ± 63.9	39.6 ± 67.5	49.5 ± 61.5	0.16
Aspartat-Aminotransferase (AST)(U/l)	34.8 ± 29.1	33.4 ± 32.6	36 ± 26.5	0.34
Cholinesterase (CHE)(U/l)	6566.8 ± 2101.9	6409.7 ± 2230.9	6701.4 ± 2016.1	0.58
Alcalic phosphatase (ALP)(U/l)	126 ± 136.1	78.6 ± 9.5	166.3 ± 174.7	0.02
Gamma glutamyl transferase (GGT)(U/l)	126.9 ± 194.6	69.8 ± 154.3	174.1 ± 194.6	0.009
Carcinoembryonic antigen (CEA) (nanogramm/liter (ng/l))	3.1 ± 2.8	2.6 ± 2.8	3.8 ± 2.7	0.07
Carbohydrate antigen (CA) 19-9 (U/l)	202.7 ± 734.0	20.3 ± 31.6	352.4 ± 972.2	0.001
Lipase (U/l)	80.1 ± 209.9	106.2 ± 311.6	59.1 ± 50.0	0.59
Amylase (U/l)	41.7 ± 80.7	55.2 ± 116.0	30.8 ± 31.0	0.19
Bilirubin (milligramm/deciliter (mg/dl))	1.2 ± 2.8	1.1 ± 1.8	1.3 ± 3.4	0.63

^a^ *SD* standard deviation

^b^ *IPMN* intraductal papillary mucinous neoplasm

**Table 3:** Pathological preoperative laboratory values

Laboratory parameter	Overall cohort (*n* = 54) mean ± SD^a^	Non-invasive IPMN^b^ mean ± SD^a^	Invasive IPMN^b^ mean ± SDa	Two-sided *p*-value
Alanin-Aminotransferase (ALT) > 45 Units/liter (U/l)	13 (25 %)	5 (21 %)	8 (28 %)	0.59
Aspartat-Aminotransferase (AST) > 35 U/l	14 (26 %)	6(25 %)	8 (28 %)	0.84
Cholinesterase (CHE) < 3900 U/l	10 (19 %)	5 (21 %)	5 (18 %)	0.79
Alcalic phosphatase (ALP) > 130 U/l	15 (30 %)	2 (8 %)	13 (48 %)	0.002
Gamma glutamyl transferase (GGT) > 55 U/l	22 (42 %)	8 (33 %)	14 (48 %)	0.28
Carcinoembryonic antigen (CEA) > 5 nanogramm/liter (ng/l)	9 (18 %)	3 (13 %)	6 (21 %)	0.45
Carbohydrate antigen (CA) 19-9 > 37 U/l	21 (41 %)	5 (22 %)	16 (57 %)	0.01
Lipase > 60 U/l	14 (26 %)	4 (17 %)	10 (33 %)	0.17
Amylase > 53 U/l	24 (44 %)	9 (38 %)	15 (50 %)	0.37
Bilirubin > 1.2 milligramm/deciliter (mg/dl)	11 (20 %)	6 (25 %)	5 (17 %)	0.46

^a^ *SD* standard deviation

^b^ *IPMN* intraductal papillary mucinous neoplasm

### Operative results

Pancreatic resection is performed as a classical Kausch-Whipple procedure in 15 %, pylorus preserving pancreaticoduodendectomy in 41 % of patients. Twenty-four percent % of patients are treated with distal and 20 % of patients with total pancreatectomy. Splenectomy is performed in 35 % of patients. The distribution of surgical approaches between invasive and non-invasive subgroups is shown in Table 4. Three patients with portal venous infiltration require a more radical surgical approach including an extended resection of mesenterico-portal venous tissue. After resection, the portal axis is reconstructed by interposition of a Gore-Tex® tube graft using a running polypropylene suture. The mean duration of surgery is 293 (range 115 – 525) minutes; fifty percent of patients require intraoperative blood products.

**Table 4:** Surgical measures

Surgical data	Overall (*n* = 54)	Non-invasive IPMN^a^ (*n* = 24)	Invasive IPMN^a^ (*n* = 30)	Two-sided *p*-value
Whipple procedure	8 (15 %)	5 (21 %)	3 (10 %)	0.27
Pylorus preserving pancreatico-duodenectomy	22 (41 %)	10 (42 %)	12 (40 %)	0.90
Distal pancreatectomy	13 (24 %)	6 (25 %)	7 (23 %)	0.89
Pancreatectomy	11 (20 %)	3 (13 %)	8 (27 %)	0.18
Vascular reconstruction	3 (6 %)	0	3 (10 %)	0.25
Intraoperative blood transfusion	27 (50 %)	10 (42 %)	17 (57 %)	0.52

^a^ *IPMN* intraductal papillary mucinous neoplasm

### Postoperative results

The median hospital stay is 23 (range 7 – 87) days, and the median ICU (intensive care unit) stay 3 (range 1 – 87) days. Twenty-six percent of all patients, 25 % of patients in the non-invasive as well as 27 % in the invasive subgroup receive transfusion of red blood cells during the postoperative period (p = 0.92). Sixty-three percent of patients show an uneventful postoperative course. Postoperative morbidity details for the other patients are displayed in detail in Table 5. Thirty-day mortality is 3.7 % (2 out of 54 pts), both with an invasive IPMN with associated carcinoma. Nineteen percent of patients, all of whom having an IPMN associated carcinoma, receive postoperative chemotherapy; none of patients undergoes radiotherapy.

**Table 5:** Postoperative morbidity details

Morbidity details	Overall (*n* = 54)
*Cholangitis*	1 (2 %)
Cholestasis	1 (2 %)
Postoperative shock	1 (2 %)
Fever of unknown origin	1 (2 %)
Anastomotic leakage	1 (2 %)
Bleeding	3 (6 %)
Pleural effusion	1 (2 %)
Abscess formation	3 (6 %)
Postoperative pneumonia	2 (4 %)
Pancreatic fistula	1 (2 %)
Multi organ failure	2 (4 %)
Atrial fibrillation	1 (2 %)

### Histopathological results

In 44 % of pts, definitive postoperative histopathological examination reveals a non-invasive IPMN. The remaining 56 % suffer from IPMN with associated carcinoma, i.e., invasive IPMN. Seven percent are classified as MD-, 17 % as BD- and 32 % as mixed type IPMN. In the remaining 44 % of patients, no further specification is undertaken. Immuno-histochemical analysis demonstrates gastral subtype in 15 %, intestinal in 17 %, pancreato-biliary in 6 % and mixed type in 11 %; in 52 %, no immuno-histochemical data are available. Details of histopathologic tumor size and localization are shown in Table 6.

**Table 6:** Histopathological details

Histology	Overall (*n* = 54)	Non-invasive IPMN^a^ (*n* = 24)	Invasive IPMN^a^ (*n* = 30)	Two-sided *p*-value
*Grade of dysplasia*				
Low grade	3 (6 %)	3 (13 %)	0	0.06
Intermediate^a^	11 (20)	11 (46 %)	0	<0.001
High grade	21 (29 %)	0	21 (70 %)	<0.001
Mixed	19 (35 %)	10 (42 %)	9 (30 %)	0.54
*Histopathological cyst size*				
<3 cm	24 (48 %)	11 (48 %)	13 (48 %)	0.91
>3 cm	26 (48 %)	12 (50 %)	14 (47 %)	0.81
Not available	4 (7 %)	1 (4 %)	3 (10 %)	0.47
R0	36 (67 %)	17 (71 %)	19 (63 %)	0.80
R1	18 (33 %)	7 (29 %)	11 (37 %)	0.68
Obstruction of the pancreatic duct	13 (24 %)	0	13 (43 %)	0.003
Concrements	2 (4 %)	0	2 (7 %)	0.21
Calcifications	8 (15 %)	3 (13 %)	5 (17 %)	0.71
Pancreatitis	30 (66 %)	14 (58 %)	16 (53 %)	0-85

^a^ *IPMN* intraductal papillary mucinous neoplasm

### Results at follow-up

Median follow-up is 42 (range 0 – 142) months. Eighty percent of patients (43 out of 54) show no evidence of disease and eleven percent are alive with disease, while the remaining seventeen percent (9 out of 54) have succumbed to their disease, all but one with a recurrence of invasive IPMN and/or IPMN with associated carcinoma [Table 7].

**Table 7:** Follow-up details

Status at follow-up	Overall (*n* = 54)	Non-invasive IPMN^a^ (*n* = 24)	Invasive IPMN^a^ (*n* = 30)	Two-sided *p*-value
No evidence of disease	43 (80 %)	23 (96 %)	20 (71 %)	0.3
Dead of disease	9 (17 %)	1 (4 %)	8 (29 %)	0.05
Alive with disease	6 (11 %)	0	6 (20 %)	0.04

^a^IPMN intraductal papillary mucinous neoplasm

**Fig. 1:**
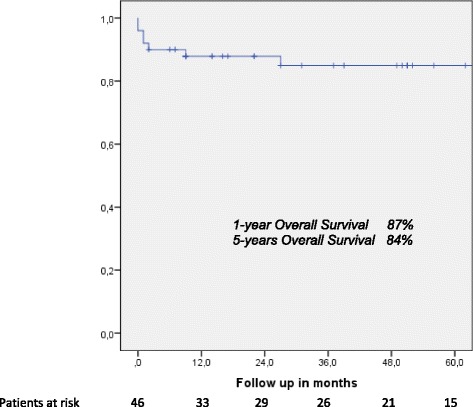
Overall one- and five-year actuarial survival

**Fig. 2:**
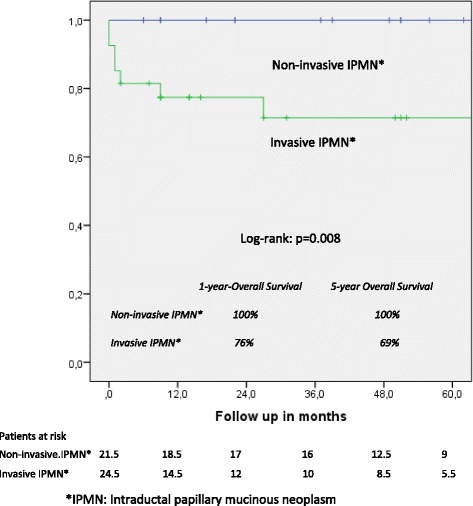
Overall survival, non-invasive vs. invasive intraductal papillary mucinous neoplasm (IPMN)

**Fig. 3:**
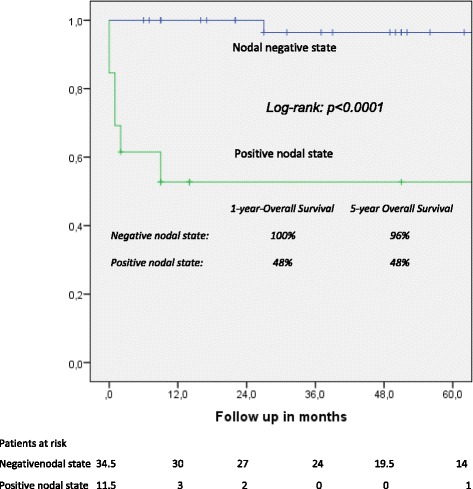
Overall survival according to nodal state

**Fig. 4:**
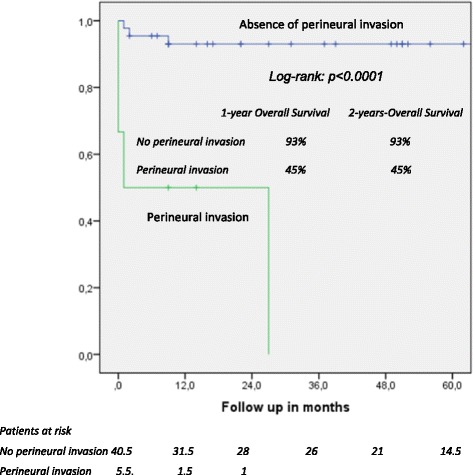
Overall survival according to lymphovascular invasion

**Fig. 5:**
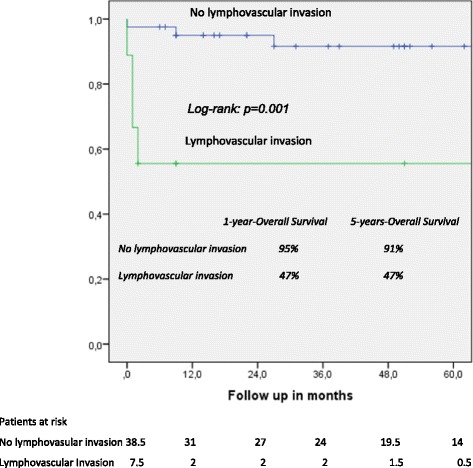
Overall survival according to perineural invasion

**FIg. 6:**
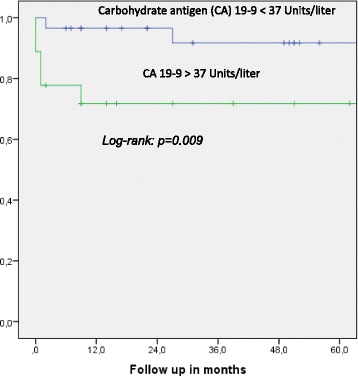
Overall survival according to preoperative Carbohydrate antigen (CA) 19-9 serum levels

**Fig. 7:**
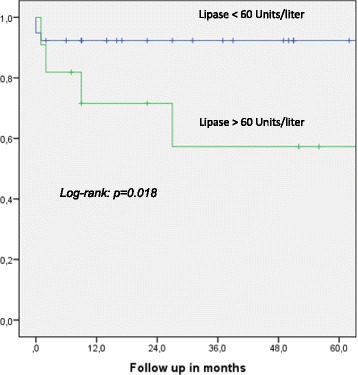
Overall survival according to preoperativ lipase serum levels

One- and five-year overall actuarial survival is 87 and 84 % for the overall cohort [Fig. 1], for non-invasive IPMN 100 % and 100 %, and for invasive IPMN 76 % and 69 %, respectively [Fig. 2]. Median overall survival is 120 months for the overall cohort; 120 months for patients with a non-invasive form and 111 months for patients with invasive IPMN. In patients with invasive IPMN, a positive nodal state, perineural invasion and lymphovascular infiltration (*p* < 0.0001 vs. *p* < 0.0001 vs. *p* = 0.001) are associated with unfavorable outcome; median overall survival in the absence of nodal disease was 120 months vs. 11.5 when IPMN is associated with nodal disease. With perineural invasion, median overall survival is 11 months vs. 120 months in the absence of same. Lymphovascular invasion is associated with a median overall survival of 11 months vs. 120 months without lymphovascular infiltration [Figs. 3, 4 and 5]. Preoperatively elevated CA 19-9 serum levels (>37 U/l) as well as elevated lipase levels (>60 U/l) are associated with unfavorable long term outcome (*p* = 0.009 vs *p* = 0.018, respectively) [Figs. 6 and 7].

**Fig. 8:**
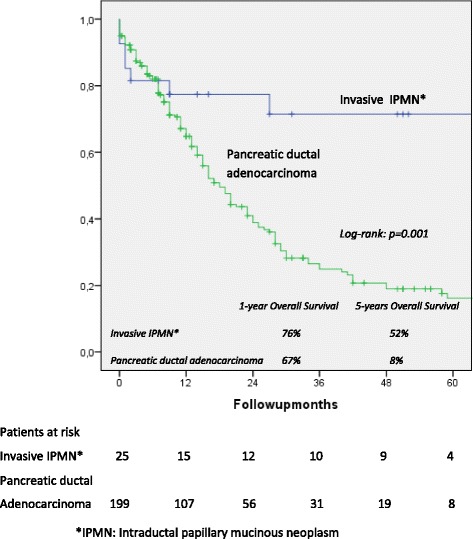
Overall survival, invasive intraductal papillary mucinous neoplasm (IPMN) vs. pancreatic ductal adenocarcinoma

Overall survival of IPMN associated carcinoma is correlated with survival of patients operated for ductal adenocarcinoma [Fig. 8], with a significantly better long-term outcome in patients undergoing pancreatic resection for IPMN associated carcinoma than for patients suffering from ductal adenocarcinoma. The median overall survival for patients with IPMN associated carcinoma is 60 months vs. 20 months for patients with ductal adenocarcinoma. There is an actuarial one- and five-year overall survival of 76 % and 52 % in patients with IPMN associated carcinoma vs. 67 % and 8 % in patients with ductal adenocarcinoma (log rank: *p* = 0.001) with no significant differences in baseline characteristics such as age, gender and comorbidities [Table 8].

**Table 8:** Baseline characteristics invasive intraductal papillary mucinous neoplasm (IPMN) vs. pancreatic ductal adenocarcinoma

Factor	Invasive IPMN^a^ (*n* = 30)	Pancreatic ductal adenocarcinoma (*n* = 221)	Two-sided *p*-value
Age (years)	67 ± 11	66 ± 9	0.58
Age > 70 years	14 (47 %)	75 (34 %)	0.17
Female gender	17 (57 %)	112 (51 %)	0.54
Body mass index	25 ± 4	26 ± 4	0.78
*American Society of Anesthesiologists (ASA) classification*			
ASA I	0	8 (4 %)	0.29
ASA II	11 (37 %)	75 (35 %)	0.77
ASA III	14 (47 %)	113 (52 %)	0.65
ASA IV	5 (17 %)	20 (9 %)	0.19

^a^ *IPMN* intraductal papillary mucinous neoplasm

## Discussion

The widespread use of imaging modalities such as MDCT and MRI has increased the frequency of incidental detection of cystic pancreatic tumors, most commonly IPMN, in patients undergoing abdominal diagnostic work-up for other diseases [19].

The distribution of age in our cohort is similar to other surveys, with a wide range from 29 to 84 years [14, 20, 21] and no preponderance of male or female gender, as in other series [19, 22].

Most of our patients are symptomatic at initial presentation, irrespective of the presence of an invasive component. The distribution of symptoms do not differ between the invasive and the non-invasive subgroup apart from diabetes mellitus, and patients in the invasive subgroup have a greater tendency toward nausea (*p* = 0.09). As also reported by D’Angelica et al., in our cohort, jaundice is not a common clinical presentation in IPMN associated carcinoma patients, as only 7 % have elevated serum bilirubin levels in contrast to 30 % of patients with ductal adenocarcinoma and higher serum bilirubin levels [15, 23].

New onset or preoperative aggravation of preexisting diabetes mellitus is more frequent in the invasive subgroup (*p* = 0.006), in agreement with Marchegiani et al. [21]. In contrast to our series, they reported significantly more patients presenting with preoperative obstructive jaundice and extensive weight loss when an invasive IPMN was present. In their series, they further differentiated between minimally invasive (<5 mm) and macroscopically invasive (>5 mm) IPMN associated carcinoma. They reported that both preoperative diabetes and obstructive jaundice were associated with macroscopic invasive carcinoma, indicating that these symptoms point to an aggressive tumor and/or locally advanced disease [22].

In our series, 56 % of resected specimens include an invasive component and/or an associated carcinoma, but there is no concomitant ductal adenocarcinoma that did not originate from premalignant intraductal lesions or PanIN. This is reflected in a significantly better median overall survival compared to median survival after resection for pancreatic ductal adenocarcinoma [23–25] as well as significantly better 5-year overall survival [23, 26]. This can be explained by slower progression of the malignant transformation into an invasive carcinoma. Interestingly, in our series, bile duct obstruction is only seen in the invasive group, with all the tumors located in the pancreatic head. This finding is supported by Brambs et al. [27, 28] as well as Ogawa et al. [29]; both judged biliary obstruction to be an index for the malignancy of an IPMN lesion [20].

As reported above, none of patients suffers from a concomitant ductal adenocarcinoma; but one-fourth of them from a current and/or previous extra-pancreatic malignancy. In four patients there is a history of renal cell carcinoma, in one each, rectal carcinoma and pulmonary carcinoma; other tumor entities included, among others, ovarian, testicular or prostate cancer, and a malignant tumor of the spine.

Sahora et al. reported a frequency of 7 % of concomitant pancreatic ductal adenocarcinoma or peri-ampullary carcinoma in a recent series of 441 patients. It mainly occurred in patients with branch duct IPMN; other synchronous neoplasms in this series were found sporadically without suspected association. This is in contrast to our data, in which most of patients exhibit anamnestic extra-pancreatic malignancy, corresponding to the life-time risk for malignancies in an age adjusted population [30, 31].

Preoperative serum levels of CA 19-9 are significantly higher in the invasive subgroup than in the non- invasive subgroup in line with the findings of Kawai et al. and Goh et al. [20, 32–35]. Nevertheless, the role of CA 19-9 as a predictor for invasiveness of an IPMN was seen critically by other authors, above all for the sub-entity of branch-duct IPMN. Sahora et al. reported that only 35 % of pts with invasive BD-IPMN carcinoma showed elevated CA 19-9 levels, while 14 % of patients with benign lesions were false positive for CA 19-9 [36], indicating that CA 19-9 is not an appropriate diagnostic tool for preoperative differentiation and decision making with respect to the potential invasiveness of a cystic pancreatic mass, but can be helpful together with cyst fluid analysis, cytopathology and radiological imaging [7, 36]. In our series, we also observe significantly elevated levels of ALP (alkaline phosphatase) and GGT (gamma glutamyltransferase) in the invasive subgroup. These laboratory values can be elevated with other pathologies such as cystic pancreatic tumors or pancreatic ductal adenocarcinoma, above all in inoperable stages, and therefore do not predict invasiveness [37].

Preoperative diagnostics in our series includes MDCT and/or MRI [20, 21, 36]. Additional ERCP is performed in 39 % and MRCP in 35 %; preoperative cytology from CT guided biopsy is available for 19 % of patients. Following implementation of the Fukuoka Guidelines, we increasingly use EUS and fine needle aspiration [7]. EUS including Doppler ultrasound provided higher diagnostic accuracy in preoperative evaluation of potential malignancy of cystic pancreatic lesions thanks to better imaging of the dilated pancreatic duct, and detailed imaging of the morphology of an intraductal lesion [1, 38]. As a limitation, there was significant inter-observer variability in the diagnostic accuracy of EUS, depending on the experience of the examiner. In experienced hands, together with PET, MRCP and CT, the malignancy of an IPMN lesion could be detected with a diagnostic accuracy of 90 % [39].

Perioperative morbidity and mortality in our series are comparable to other studies [14, 21, 22, 35, 37, 40], with no mortality in the non-invasive cohort [Table 9]. One-, five- and ten-year overall survival rates are excellent for the overall cohort but with a significantly poorer outcome in the invasive subgroup, comparable to results from other surveys [14, 22, 41]. For our non-invasive subgroup, the 10-year overall survival rate is 80 %, which is comparable to the age-adjusted attrition rate [42]. In our cohort, the recurrence rate in the non-invasive cohort is low, with one patient developing an invasive recurrence and consecutively succumbs to his disease during follow-up. All the other patients are free of tumor recurrence at follow-up. Other series reported recurrence rates up to 17 % for resected non-invasive IPMN [36, 43, 44]. In our series, after resection of invasive IPMN seventy-nine percent of patients are free of tumor recurrence during follow-up. In other series, the reported recurrence rate was significantly higher, with 28 – 60 % after pancreatic resection for invasive IPMN [20, 40, 45].

**Table 9:** Perioperative and long-term outcome after pancreatic resection for intraductal papillary mucinous neoplasm (IPMN)

Series	number of patients	non-invasive IPMN^a^ (n/%)	Invasive IPMN^a^ (n/%)	Perioperative mortality (*n*/%)	Perioperative morbidity (Clavien Dindo/others; *n*(%))	Survival	Recurrence rate	Comment
Salvia et al. [44]	*n* = 140	47 (34 %)	83 (66 %)	0	33 (24 %)(major complication)	10-year overall survival non-invasive IPMN^a^ 100 %; invasive IPMN^a^: 50 %.	n.s.^b^	
Sohn et al. [14]	*n* = 136	84 (62 %)	52 (38 %)	5 (3.7 %)	47 (35 %)	5-year overall survival non-invasive IPMN^a^ :77 %; invasive. IPMN^a^: 43 %.	n.s.^b^	
D'Angelica et al. [15]	*n* = 62 ^a^	33^a^ (52 %)	30 (48 %)	4 (6 %)	31 (50 %)	5-year-overall survival:75 %/10 year-overall survival:60 %	23 %	^a^ 1 patient Unresectable
Rodriguez et al. [39]	*n* = 145	113 (78 %)	32 (22 %)	0	86 (59 %)	10-year-overall survival: 70 %, invasive Carcinoma : 10-year-overall survival: 63 %	6.9 %	only branch duct-IPMN^a^
Niedergethmann [20]	*n* = 97	29 (30 %)	68 (70 %)	1 (1 %)	55 (56.7 %)	median overall survival: 36 months	n.s.^b^	
Sahora et al. [35]	*n* = 226	174 (77 %)	52 (23 %)	1.3 %	34 %	n.s.	8.5 %	only branch-duct-IPMN^a^
Marchegiani et al. [21]	*n* = 173	48 (28 %)	125 (72 %)	0	15.6 % ("major surgical complications")	10 year-overall survival: 69 %	25 %	only main-duct-IPMN^a^
present series	*n* = 54	24 (44 %)	30 (56 %)	2 (3.7 %)	20 (37 %)	5-year-overall survival non-invasive IPMN^a^: 100 %, invasive IPMN^a^ 69 %	6 (12 %)	

^a^ *IPMN* intraductal papillary mucinous neoplasm

^b^ *n.s.* not specified

An invasive component, a positive nodal state, and lymphovascular and perineural invasion are associated with a significantly poorer outcome in our study. Lymph node positivity was also predictive for lower long-term survival rates in other series [14, 22]. As is known for pancreatic ductal adenocarcinoma, lympho-vascular as well as perineural invasion were histological surrogates for advanced disease and therefore associated with unfavorable long-term outcome [25, 26, 45, 46].

The Fukuoka Guidelines offer a useful orientation on the treatment of IPMN, but even with the high diagnostic accuracy of radiologic imaging techniques, EUS, FNA, cyto-pathological, immune-histochemical and genetic analyses, cystic pancreatic lesions cannot always be classified correctly before resection. On the one hand, we have to be aware of the potential danger that a cystic lesion classified as low risk can progress to an invasive carcinoma over time. Treatment options should be thoroughly discussed with every patient with a cystic pancreatic mass and weighed carefully against a watchful waiting strategy, particularly with young, fit patients with low perioperative risk. On the other hand, we are encouraged to follow the therapeutic algorithm of the Fukuoka Guidelines [7] and avoid “unnecessary” pancreatic resections that could result in even higher mortality that would then be IPMN-associated [19]. This is of special impact for patients with diffuse distribution of low-risk IPMN where curative resection is only accessible by means of total pancreatectomy – we performed total pancreatectomy in 13 % of patients with non-invasive IPMN – with postoperative brittle diabetes and its associated risks and reduced quality of life as a consequence [22].

### Limitations and strength of the study

This report has all the limitations of a retrospective single center study. Because of the retrospective nature of this study over a 13-year period, numerous patients in this series were not treated with strict adherence to the Fukuoka guidelines. We cannot exclude that some patients in the non-invasive subgroup were over-treated from the present point of view. Our study deals with a patient population suffering from what is still a challenging disease that demands a “tailored approach“, taking into account both the guidelines and the individual patient’s state of health. This is indispensable if surgeons are to choose an optimal medical treatment that would neither miss potentially invasive IPMN nor over-treat, with subsequent surgery-related morbidity and mortality.

## Conclusion

Both long-term and disease-free survival after pancreatic resection for non-invasive IPMN is excellent; survival rates after pancreatic resection for invasive IPMN and IPMN-associated carcinoma are significantly higher than for patients undergoing resection for pancreatic ductal adenocarcinoma. In low- and intermediate risk IPMN with no clear indication for surgical intervention, a close follow-up strategy according to the guidelines [7] should be considered carefully and evaluated against surgical treatment for every potential risk candidate risk by an interdisciplinary board consisting of hepatobiliary surgeons, pathologists, radiologists and oncologists.

## Abbreviations

ALP: Alcalic phosphatase
BD-IPMN: Branch-duct IPMN
CA 19-9: Carbohydrate antigen
CEA: Carcino-embryonic antigen
CT: Computed tomography
ERCP: Endoscopic retrograde cholangio-pancreaticography
EUS: Endoscopic ultrasound
FNA: Fine needle aspiration
GGT: Gamma glutamyl transferase
ICU: Intensive care unit
IPMN: Intraductal papillary mucinous neoplasm
MDCT: Multi-detector computed tomography
MD-IPMN: Main-duct IPMN
MRCP: Magnetic resonance cholangio-pancreaticography
MRI: Magnetic resonance imaging
PanIN: Pancreatic intraepithelial neoplasia
PET: Positrone emission tomography
WHO: World Health Organization
